# Sex-specific associations of serum cortisol with brain biomarkers of Alzheimer’s risk

**DOI:** 10.1038/s41598-024-56071-9

**Published:** 2024-03-06

**Authors:** Lisa Mosconi, Schantel Williams, Caroline Carlton, Camila Zarate, Camila Boneu, Francesca Fauci, Trisha Ajila, Matilde Nerattini, Steven Jett, Caroline Andy, Michael Battista, Silky Pahlajani, Joseph Osborne, Roberta Diaz Brinton, Jonathan P. Dyke

**Affiliations:** 1 Department of Neurology, Weill Cornell Medicine, New York, NY 10021 USA; 2 Department of Radiology, Weill Cornell Medicine, New York, NY USA; 3 Department of Population Health Sciences, Weill Cornell Medicine, New York, NY USA; 4 Department of Clinical Pathophysiology, Nuclear Medicine Unit, University of Florence, Florence, Italy; 5 Department of Neurology and Pharmacology, University of Arizona, Tucson, AZ USA

**Keywords:** Cognitive ageing, Stress and resilience, Predictive markers, Neurological disorders, Dementia, Neurodegenerative diseases

## Abstract

Emerging evidence implicates chronic psychological stress as a risk factor for Alzheimer’s disease (AD). Herein, we examined the relationships between serum cortisol and multimodality brain AD biomarkers in 277 cognitively normal midlife individuals at risk for AD. Overall, higher cortisol was associated with lower total brain volume, lower glucose metabolism (CMRglc) in frontal cortex, and higher β-amyloid (Aβ) load in AD-vulnerable regions; and marginally associated with phosphocreatine to ATP ratios (PCr/ATP) in precuneus and parietal regions. Sex-specific modification effects were noted: in women, cortisol exhibited stronger associations with Aβ load and frontal CMRglc, the latter being more pronounced postmenopause. In men, cortisol exhibited stronger associations with gray matter volume and PCr/ATP measures. Higher cortisol was associated with poorer delayed memory in men but not in women. Results were adjusted for age, Apolipoprotein E (APOE) epsilon 4 status, midlife health factors, and hormone therapy use. These results suggest sex-specific neurophysiological responses to stress, and support a role for stress reduction in AD prevention.

## Introduction

Emerging evidence implicates chronic psychological stress as a risk factor for AD^1,2^. Stress activates the hypothalamic–pituitary–adrenal (HPA) axis prompting a glucocorticoid cascade, the main end-product of which is a raise in cortisol levels^3^. In animal studies, high glucocorticoid levels increase beta-amyloid (Aβ) deposition and tau pathology^4,5^, as well as neuronal loss in AD-vulnerable areas rich in glucocorticoid receptors (GR), such as medial temporal lobes and frontal cortex^6–9^. Cortisol also regulates several cardiovascular, metabolic, immunological and homeostatic pathways in brain, and chronic stress has been associated with hypertension, metabolic syndrome and a compromised immune systemy^10^, which are risk factors for AD in turn^11^.

High cortisol levels have been linked to hippocampal atrophy, reduced cerebral glucose metabolism (CMRglc), and dementia severity in AD patients^1,2^. Additionally, elevated cortisol has been linked with an increased risk of AD and dementia^12–15^, higher Aβ load^16,17^, smaller global and regional gray matter volumes and poorer cognitive functioning in the elderly^18–22^. In a study of midlife individuals, cortisol's impact on brain volumes and memory performance was more pronounced in women than age-controlled men^23^. This is relevant given the higher prevalence of AD in women^24^, with postmenopausal women constituting over 60% of all those affected. Women are also more susceptible to stress-related psychiatric disorders, such as post-traumatic stress disorder and clinical burnout syndrome^25^. Notably, the decline in gonadal steroid hormones, especially 17β-estradiol, during menopause has been implicated as a potential female-specific risk factor for AD^26–29^, while also affecting stress-related neural networks and HPA axis function^30^. In mechanistic analyses, menopause exerts its actions on AD risk via alterations of multiple neurobiological mechanisms that can span decades^26^, thus overlapping with the prodromal phase of the disease^31^.

Currently, no studies have examined the associations between cortisol levels and biomarkers of AD pathology in midlife, when the potential for AD prevention is greatest, or examined how these associations vary by sex and menopausal status.

Herein, we examined the relationships between serum cortisol levels and a panel of multimodality brain imaging AD biomarkers [Aβ load assessed by ^11^C-Pittsburgh compound B (PiB) Positron Emission Tomography (PET), gray matter volume assessed with Magnetic Resonance Imaging (MRI), CMRglc on ^18^F-fluorodeoxyglucose (FDG) PET, and adenosine triphosphate (ATP) production measured via ^31^Phosphorus Magnetic Resonance Spectroscopy (^31^P-MRS)], as well as cognitive performance, in asymptomatic midlife individuals at risk for AD. We then tested whether these associations differed based on sex and menopause status.

## Results

### Participant characteristics

Three-hundred and nine individuals were enrolled in this multimodality imaging study. Thirty participants were excluded for the following reasons: 24 completed imaging before cortisol analysis was added to the protocol, 7 had incidental findings (1 aneurysm, 2 meningiomas, 1 demyelination consistent with possible MS, 1 cerebellar infarct, 1 lacunar infarct), and 1 MRI scan was excluded due to artifacts. A total of 277 individuals with complete cortisol and volumetric MRI were included in analysis. Four did not complete the MRS scan due to claustrophobia or technical issues. PET imaging was done on 147 (53%) of participants.

Participant characteristics are found in Table 1. The cohort was 79% female, with mean age 51 (SD = 7) years. The mean serum cortisol level was 11 (SD = 5), range 2–32 μg/dL. APOE-4 carrier status was found in 44% of individuals. Participants were in good general health, with a small percentage of individuals diagnosed with medically controlled diabetes (2%), hypercholesterolemia (7%), hypertension (9%), and/or a history of mild depression (16%). Among women, 40% were postmenopausal, including 8% in surgical menopause. Twenty-two percent reported taking menopause hormone therapy (HT). There were no sex differences in demographic or clinical measures except for a higher presence of medically-controlled hypertension in men compared to women (*P* = 0.007, Table 1).

**Table 1:** Participant characteristics.

	Overall	Men	Women
N	277	57	220
Cortisol, μg/dL	11.1 (4.7)	11.0 (3.0)	11.1 (4.8)
Age, years	51 (7)	51 (3)	51 (6)
Education, years	17 (2)	18 (2)	17 (2)
MoCA scores, unitless	28 (2)	29 (1)	28 (2)
Race, % white	81	75	82
APOE-4 status, % carrier	44	53	42
Smoking, % ever	22	19	24
Hypertension, %	9	19	6*
Diabetes, %	2	5	1
Hypercholesterolemia, %	7	7	6
Depression history, %	16	12	17
Postmenopausal status, %	–	–	40
Oophorectomy status, %	–	–	8
Menopause hormone therapy, % user	–	–	22

Mean (standard deviation) unless otherwise specified. *P < 0.05.

APOE-4, apolipoprotein E (APOE) epsilon 4; MoCA, Montreal cognitive assessment.

### Principal component analysis (PCA)

Supplementary Table 1 displays the composition of extracted factors (all loading coefficients > 0.55), which accounted for ≥ 68% of the total variance in the biomarker panel for each modality. For brain volumes and PCr/ATP, Factor 1 included frontal, temporal and posterior cingulate regions and Factor 2 included precuneus and parietal regions. For CMRglc, Factor 1 included mainly parietal regions, posterior cingulate and precuneus, Factor 2 included superior frontal and various temporal regions, and Factor 3 included mainly middle frontal regions. Modality-specific standardized scores from each of these factors were carried into hypothesis testing.

### Associations between serum cortisol and brain biomarkers

**Figure 1:**
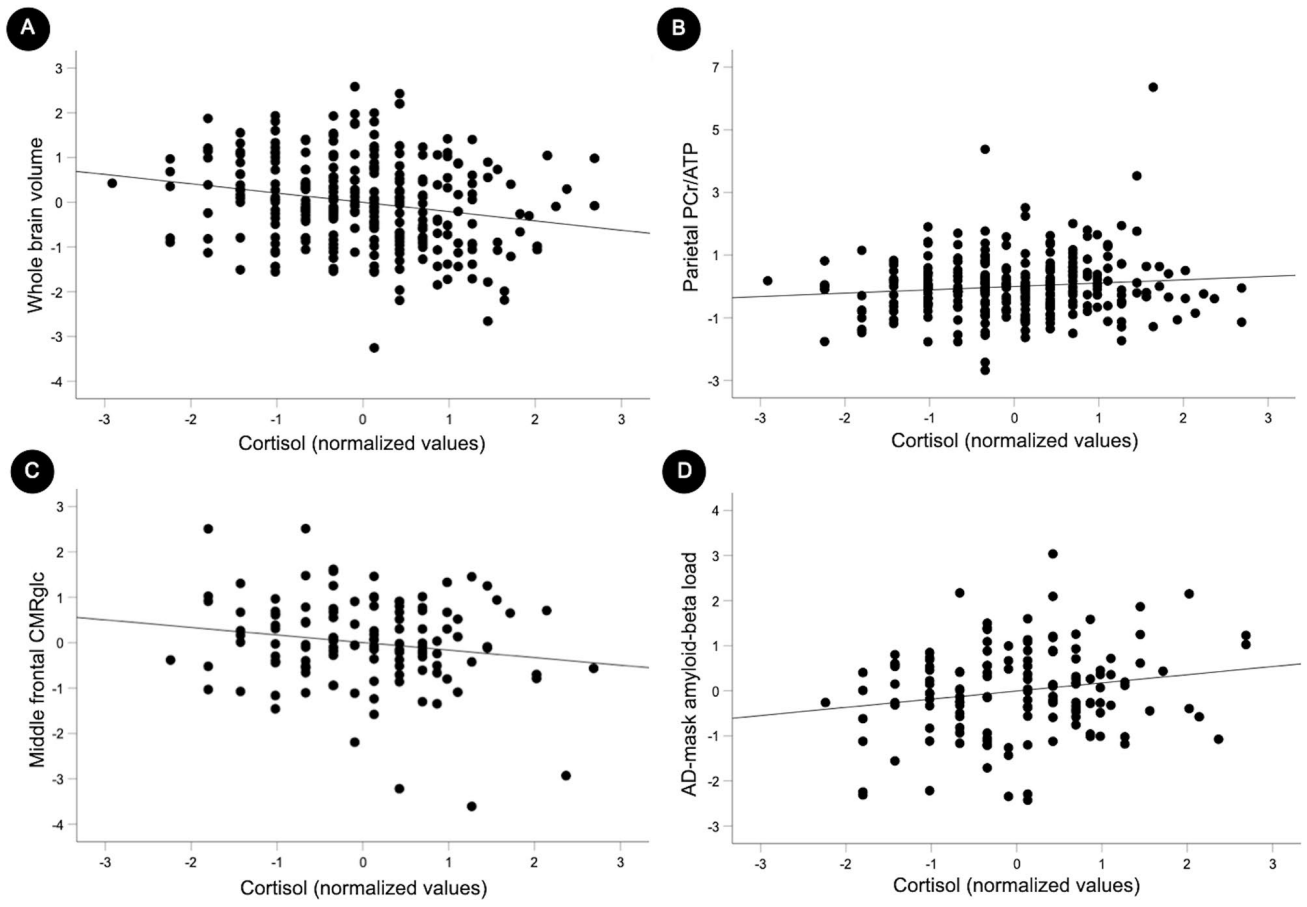
Associations of serum cortisol with brain biomarkers. Scatterplots showing associations between cortisol and brain biomarkers: (**A**) total brain volume. (**B**) Phosphocreatine to ATP ratio (PCr/ATP) in precuneus and parietal regions (Factor 2 from principal component analysis of ^31^P-MRS data). (**C**) Cerebral metabolic rates of glucose (CMRglc) in middle frontal regions (Factor 3 from principal component analysis of ^18^F-FDG PET data). (**D**) Amyloid-beta (Aβ) load in AD-mask. Analyses are multivariable-adjusted by age, APOE-4 status, midlife health variables, and modality specific confounders. Cortisol measures underwent a standardized asinh(x) transformation prior to analysis. Standardized values are displayed in the graphs.

Results are summarized in Table 2. Cortisol exhibited negative associations with total brain volume (β [SE]: − 0.081 [0.023], multivariable adjusted *P* < 0.001, Fig. 1A) and with CMRglc in middle frontal gyrus (PCA Factor 3; β [SE]: − 0.196 [0.099], multivariable adjusted *P* = 0.038, Fig. 1B). Conversely, cortisol exhibited positive associations with Aβ load in AD-mask (β [SE]: 0.194 [0.086], multivariable adjusted *P* = 0.023; Fig. 1C) and marginal positive associations with PCr/ATP in precuneus and parietal regions (PCA Factor 2; β [SE]: 0.120 [0.062], multivariable adjusted *P* = 0.053; Fig. 1D).

**Table 2:** Associations of cortisol with brain biomarkers.

	Model 1			Model 2		
	Coeff.	SE	P	Coeff.	95% C.I.	P
Brain volume						
Total volume	− 0.077	0.022	** < 0.001**	− 0.081	0.023	** < 0.001**
FAC1_frontotemporal_	− 0.036	0.046	0.434	− 0.039	0.047	0.404
FAC2_parietal_	− 0.052	0.054	0.325	− 0.051	0.055	0.350
PCr/ATP						
FAC1_frontotemporal_	0.046	0.060	0.445	0.036	0.060	0.542
FAC2_parietal_	0.115	0.060	**0.059**	0.120	0.062	**0.053**
CMRglc						
FAC1_parietal_	− 0.025	0.041	0.683	− 0.048	0.097	0.605
FAC2_frontotemporal_	− 0.049	0.042	0.426	− 0.087	0.100	0.361
FAC3_middlefrontal_	− 0.129	0.042	**0.034**	− 0.196	0.099	**0.038**
Aβ load						
AD-mask	0.132	0.044	**0.031**	0.194	0.086	**0.023**

Standardized beta coefficients and standard errors (SE) from regression models adjusted by age, gender, APOE4 status (Model 1); and midlife health variables (Model 2). Significant *P* values are in bold.

Brain volumes are adjusted by total intracranial volume. ^31^P-MRS measures are phosphocreatine (PCr) to adenosine triphosphate (ATP) ratios. Cerebral metabolic rates of glucose (CMRglc) and amyloid-β (Aβ) positron emission tomography (PET) measures are standardized to cerebellar gray matter uptake. N = 147 participants had PET scans.

### Associations between serum cortisol and brain biomarkers by sex

**Figure 2:**
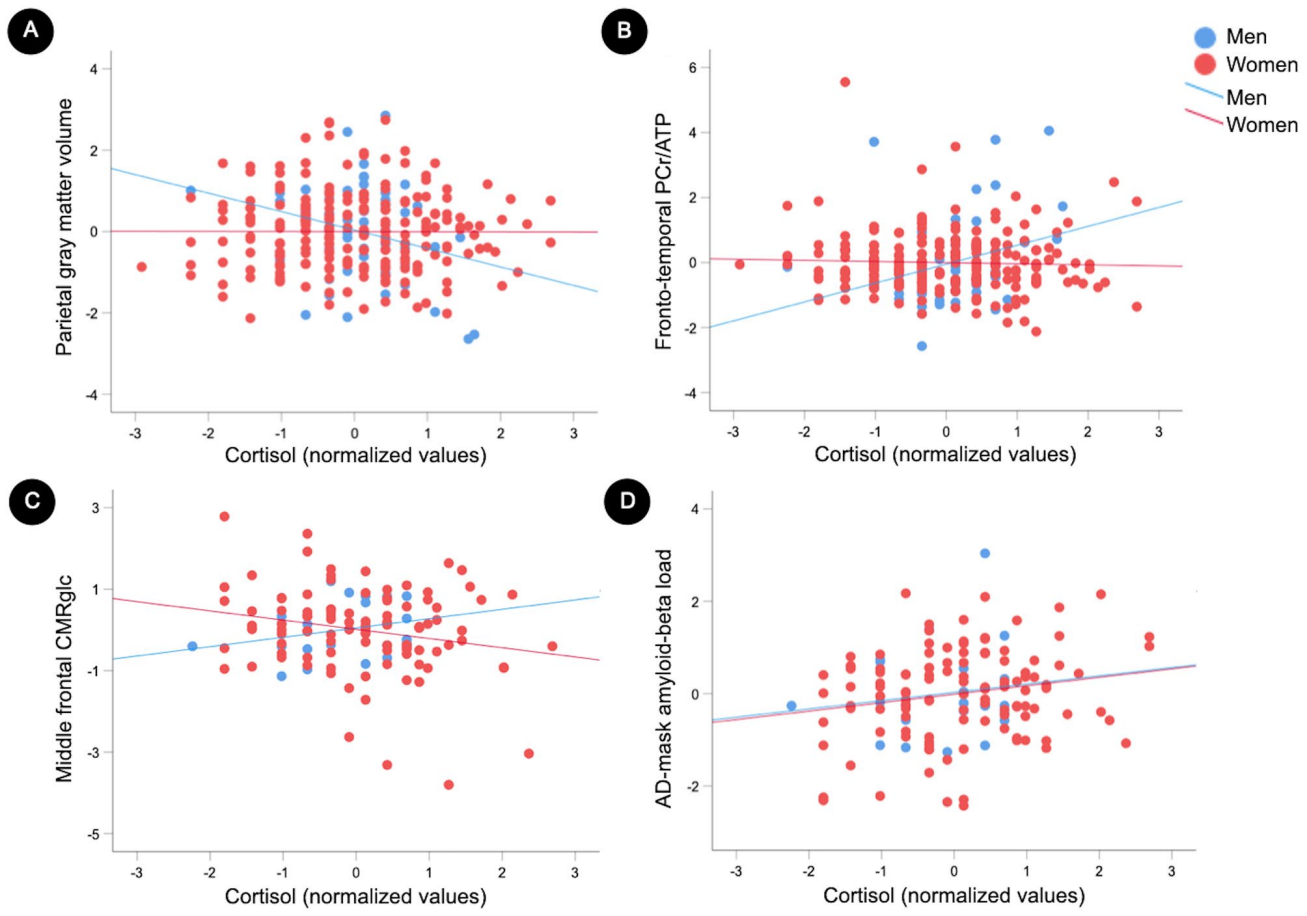
Associations of serum cortisol with brain biomarkers by sex. Scatterplots showing sex effects on the associations of cortisol with brain biomarkers: (**A**) Gray matter volume in precuneus, inferior and superior parietal regions (Factor 2 from principal component analysis of MRI data). (**B**) Phosphocreatine to ATP ratio (PCr/ATP) in frontal, temporal and posterior cingulate regions (Factor 1 from principal component analysis of ^31^P-MRS data). (**C**) Cerebral metabolic rates of glucose (CMRglc) in middle frontal regions (Factor 3 from principal component analysis of ^18^F-FDG data). (**D**) Amyloid-beta (Aβ) load in AD-mask. Analyses are multivariable-adjusted by age, APOE-4 status, midlife health variables, and modality specific confounders. Cortisol measures underwent a standardized asinh(x) transformation prior to analysis. Standardized values are displayed in the graphs. Men = blue; women = red. In (**A**–**C**), cortisol-biomarker associations demonstrate a differential effect of cortisol on these outcomes by sex, as evidence by significant interaction terms. Conversely, in (**D**), no sex-based modification effects were detected in the associations between cortisol and Aβ. Corresponding statistics are reported in Table 3.

Results are summarized in Table 3. Sex-based modification effects were observed in the associations between cortisol and volume in precuneus and parietal regions [PCA Factor 2] (multivariable adjusted *P*_interaction_ = 0.035), which were driven by presence of significant associations for men (*P* = 0.017) and not for women (*P* = 0.779) (Fig. 2A). Similarly, sex by cortisol interactions were observed in the associations between cortisol and PCr/ATP in frontal, temporal and posterior cingulate regions [PCA Factor 1] (multivariable adjusted *P*_interaction_ = 0.001). In these regions, associations of cortisol with PCr/ATP were significant for men (*P* = 0.004) and not among women (*P* = 0.597) (Fig. 2B).

**Table 3:** Associations of cortisol with brain biomarkers by sex.

	Men						Women						Interaction P
	Model 1			Model 2			Model 1			Model 2			
	Coeff.	SE	P	Coeff.	SE	P	Coeff.	SE	P	Coeff.	SE	P	
Brain volume													
Total volume	− 0.115	0.063	0.071	− 0.129	0.063	**0.038**	− 0.092	0.030	**0.001**	− 0.097	0.031	**0.001**	0.242
FAC1_frontotemporal_	0.061	0.187	0.600	0.059	0.193	0.637	− 0.061	0.047	0.267	− 0.066	0.048	0.244	0.341
FAC2_parietal_	− 0.324	0.189	**0.010**	− 0.322	0.204	**0.017**	− 0.016	0.055	0.796	− 0.018	0.057	0.779	**0.035**
PCr/ATP													
FAC1_frontotemporal_	0.361	0.229	**0.007**	0.379	0.223	**0.004**	− 0.031	0.056	0.654	− 0.038	0.058	0.597	**0.001**
FAC2_parietal_	0.259	0.218	0.061	0.180	0.216	0.188	0.106	0.062	0.126	0.105	0.064	0.141	0.236
CMRglc													
FAC1_parietal_	− 0.046	0.192	0.846	0.306	0.167	0.167	− 0.021	0.107	0.834	− 0.006	0.110	0.956	0.724
FAC2_frontotemporal_	− 0.026	0.153	0.914	0.361	0.128	0.097	− 0.122	0.114	0.252	− 0.116	0.119	0.297	0.442
FAC3_middlefrontal_	0.280	0.192	0.228	0.548	0.195	0.054	− 0.251	0.110	**0.016**	− 0.238	0.114	**0.026**	**0.032**
Aβ load													
AD-mask	0.118	0.122	0.403	0.062	0.345	0.826	0.116	0.048	0.097	0.192	0.092	**0.049**	0.467

Standardized beta coefficients and standard errors (SE) from regression models adjusted by age and APOE4 status (Model 1); and after further adjustment by midlife health variables for both sexes, and by hormone therapy use for women (Model 2). Significant *P* values are in bold. Brain volumes are adjusted by total intracranial volume. ^31^P-MRS measures are phosphocreatine (PCr) to adenosine triphosphate (ATP) ratios. Cerebral metabolic rates of glucose (CMRglc) and amyloid-β (Aβ) positron emission tomography (PET) measures are standardized to cerebellar gray matter uptake. N = 147 participants had PET scans.

Sex-based modification effects were also observed in the associations between cortisol and CMRglc in middle frontal regions [PCA Factor 3] (multivariable adjusted *P*_interaction_ = 0.032). Sex-stratified analysis showed differential cortisol-CMRglc association patterns, with negative associations in women (β [SE]: − 0.238 [0.114], *P* = 0.026) and non-significant positive associations in men (β [SE]: 0.548 [0.195], multivariable adjusted *P* = 0.054) (Fig. 2C).

There were no cortisol by sex interaction effects on Aβ load in AD-mask (Fig. 2D), although cortisol-Aβ associations were significant in women (β [SE]: 0.192 [0.092], multivariable adjusted *P* = 0.049) and not in men (β [SE]: 0.062 [0.345], multivariable adjusted *P* = 0.826) (Table 3).

### Associations between cortisol and cognitive measures

As shown in Table 4, there were generally negative, albeit non-significant associations between serum cortisol and cognitive scores in the entire cohort. Sex-based modification effects were observed in the relationship between cortisol and RAVLT total scores (multivariable adjusted *P*_interaction_ = 0.035) (Supplementary Table 2). These effects were driven by men exhibiting stronger associations of cortisol levels with RAVLT total scores (β [SE]: − 0.318 [0.207], multivariable adjusted *P* = 0.025) compared to women (β [SE]: − 0.018 [0.065], multivariable adjusted *P* = 0.796) (Supplementary Table 2).

**Table 4:** Associations of cortisol with cognitive performance.

	Model 1			Model 2		
	Coeff.	SE	P value	Coeff.	SE	P value
Logical memory, immediate	− 0.017	0.062	0.784	− 0.028	0.062	0.651
Logical memory, delayed	0.026	0.062	0.667	0.015	0.061	0.804
RAVLT, total	− 0.036	0.062	0.557	− 0.047	0.062	0.446
RAVLT, delayed	− 0.095	0.061	0.119	− 0.099	0.061	0.103
RAVLT, recognition	− 0.054	0.062	0.377	− 0.048	0.062	0.433
FAS (fluency)	0.015	0.064	0.807	0.012	0.063	0.841
Trail making test-B	− 0.091	0.064	0.149	− 0.073	0.065	0.252

Standardized beta coefficients and standard errors (SE) from regression models adjusted by age and education (Model 1); and further by gender and APOE-4 status (Model 2).

RAVLT, Rey auditory verbal learning test.

### Sensitivity analysis

#### Associations between cortisol and regional biomarker measures

**Figure 3:**
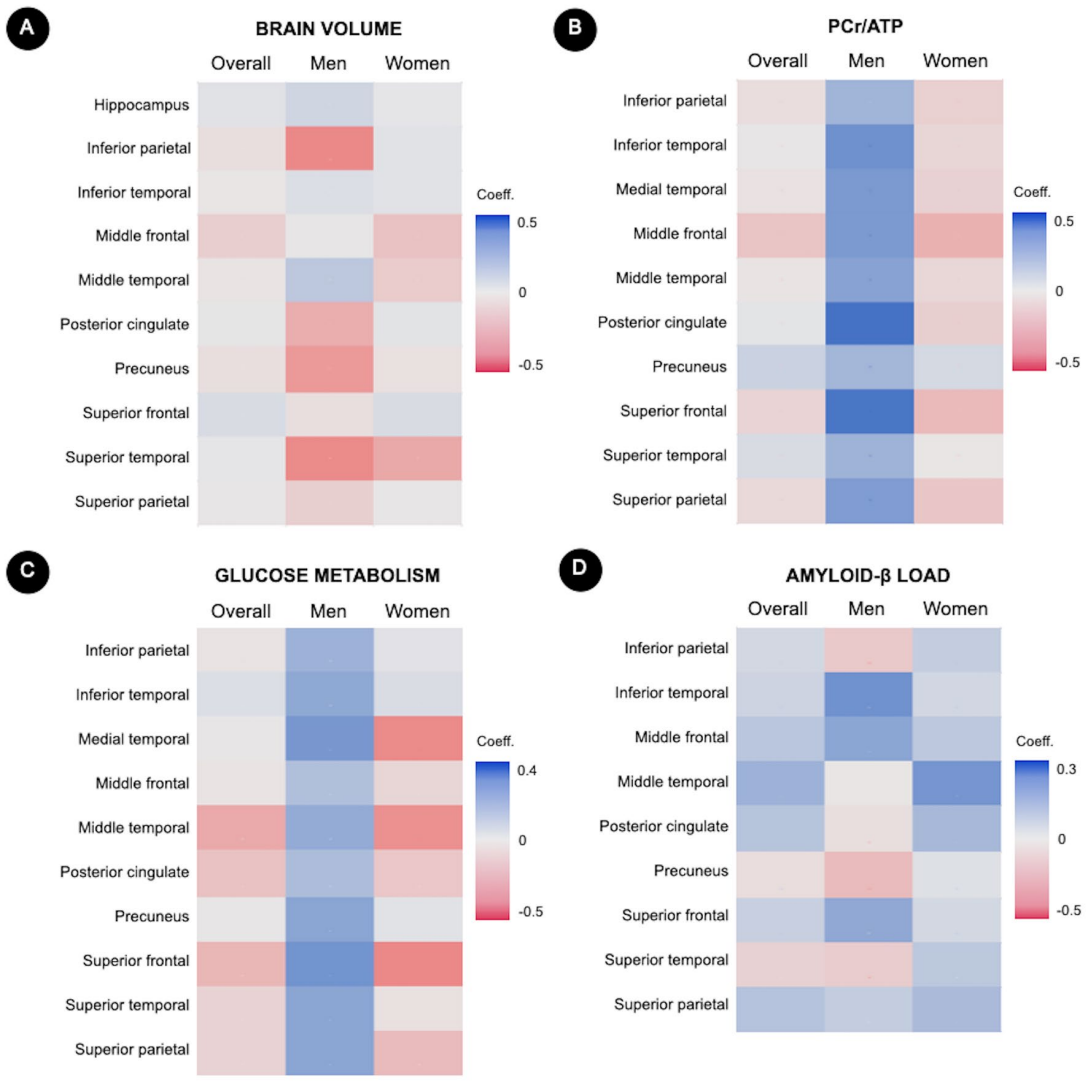
Associations between serum cortisol and regional biomarker measures on a region-by-region basis. Heatmaps showing associations between cortisol levels and (**A**) regional brain volumes, (**B**) PCr/ATP, (**C**) glucose metabolism, and (**D**) Aβ load in individual regions of interest. Multivariable-adjusted regression coefficients are displayed on a red-to-blue color-coded scale, with red indicating negative associations and blue indicating positive associations. Corresponding statistics are found in Supplementary Table 2.

To examine which regions were involved in the above effects, individual ROI results are shown in Fig. 3 and Supplementary Table 3, for descriptive purposes.

In analysis of brain volumes, there were no significant sex-based modification effects. Descriptively, cortisol levels were associated with superior parietal and inferior parietal volume in men but not in women (Supplementary Table 3 and Fig. 3A), which provides insight into the sex differences identified in PCA Factor 2 volume measures. Additionally, cortisol was associated with middle frontal volume in women but not in men (Supplementary Table 3 and Fig. 3A). In analysis of regional PCr/ATP, men exhibited stronger cortisol-biomarker associations in posterior cingulate, superior and middle frontal, inferior and medial temporal regions, compared to women (*P*_*interaction*_ < 0.05, corrected for multiple comparisons) (Supplementary Table 3 and Fig. 3B). Conversely, associations between cortisol and regional CMRglc were generally negative in women and varied from neutral to positive in men (Fig. 3C), although the interaction terms were not significant after adjusting for multiple comparisons (Supplementary Table 3). As in the main analysis, there were no significant cortisol by sex interactions on Aβ load (Fig. 3D, Supplementary Table 3).

#### Effects of menopausal status

Participant characteristics by menopause status are shown in Supplementary Table 4. No significant age differences were observed between the postmenopausal and perimenopausal groups, or compared to men. Both menopausal groups showed a lower frequency of medically-controlled hypertension compared to men (*P* = 0.05). Results from the fully adjusted models are presented below.

Supplementary Table 5 summarizes results of sex-based modification effects by menopause status. For middle frontal CMRglc [PCA Factor 3], we observed sex-based interactions for comparisons between men and postmenopausal women (multivariable adjusted *P*_*interaction*_ = 0.024), but not between men and perimenopausal women (multivariable adjusted *P*_*interaction*_ = 0.179). On post-hoc analysis, the postmenopausal group exhibited negative cortisol-CMRglc associations in middle frontal regions (postmenopause β [SE]: − 0.194 [0.092], *P* = 0.039), while men showed non-significant positive associations (β [SE]: 0.548 [0.195], *P* = 0.054).

Menopause status did not impact the sex-based associations of cortisol with PCr/ATP across frontal, temporal and posterior cingulate regions [PCA Factor 1], as interaction effects were significant in both comparisons—men vs. postmenopausal women (multivariable adjusted *P*_*interaction*_ = 0.001) and men vs. perimenopausal women (multivariable adjusted *P*_*interaction*_ = 0.009) (Supplementary Table 5).

Similarly, no significant effect of menopause status was observed on the sex-based associations of cortisol with volume in precuneus and parietal regions [PCA Factor 2]. In these regions, interaction terms were non-significant but exhibited a similar magnitude in comparisons between men and postmenopausal women (multivariable adjusted *P*_*interaction*_ = 0.063), and men and perimenopausal women (multivariable adjusted *P*_*interaction*_ = 0.092) (Supplementary Table 5).

#### Mediation analysis: associations between biomarkers and cognition

There were no overall associations between biomarkers and RAVLT scores. On sex-stratified analysis, RAVLT scores were negatively associated with PCr/ATP in frontal, temporal and posterior cingulate regions [PCA Factor 1] in men (β [SE]: − 0.238 [0.102], *P* = 0.023), and positively associated with PCr/ATP in precuneus and parietal regions [Factor 2] in women (β [SE]: 0.142 [0.070], *P* = 0.042).

## Discussion

In this multimodality brain imaging study of midlife individuals at risk for AD, higher serum cortisol concentrations were associated with lower total brain volume, lower CMRglc in frontal cortex, and higher Aβ load in a composite AD-mask, and marginally associated with brain energy production as reflected in PCr/ATP ratios. These association patterns varied by sex and to some extent, by menopause status, and were independent of age, APOE4 status, midlife health factors, and HT usage.

A growing body of evidence indicates that increased cortisol at the presymptomatic and early clinical stages of AD is associated with poorer prognosis and more rapid cognitive decline^12–15,32^. However, studies combining cortisol measures with AD biomarkers and cognitive assessments are limited, especially among cognitively normal individuals. Most prior imaging studies utilized MRI-derived brain volumes as the primary outcome measures, focusing on hippocampus and limbic areas which are involved in regulating the HPA axis and are particularly vulnerable to neuronal damage in AD^31^. However, GRs are expressed throughout the brain^6–9^, including cortical regions with known pathological and metabolic vulnerability to AD, such as frontal and cingulate cortex^31^. In keeping with GR distribution, some studies reported associations between higher serum cortisol and lower volume in whole brain^18,23^, hippocampus^19–22^, and frontal, temporal and parietal cortices^22,23^, while others reported no associations^33,34^. Notably, two PET investigations reported a link between higher serum cortisol and brain Aβ load in non-demented elderly, MCI and AD patients^16,17^. A few studies also indicated possible mediating effects of brain volume on the cognitive changes associated with cortisol levels^22,33,34^ as well as cortisol-mediated effects of Aβ on cognition in the elderly^17^.

To our knowledge, there is only one prior study investigating cortisol’s impact on brain biomarkers in midlife^23^. This study examined 1.5 Tesla MRI scans from the community-based Framingham Heart Study, showing that higher serum cortisol was associated with lower total brain volume, frontal and parietal volumes, as well as lower white matter integrity and poorer memory in middle-aged adults^23^. In secondary analyses, these associations were more pronounced in women than in men^23^. As declines in neocortical volume and white matter integrity are not specific to AD, it remains to be established whether these changes were due to AD or other causes.

Our findings expand on existing literature in several ways. First, we explored the associations of serum cortisol levels with a panel of established brain AD biomarkers, including Aβ load—the main pathological hallmark of AD—and cognitive measures, which enabled testing of mediation effects. Secondly, unlike previous studies that did not specifically target at-risk individuals, our cohort consists of individuals with a high a priori risk of AD, as indicated by a family history of AD and/or presence of the APOE4 genotype. This focus enhances the statistical likelihood that the observed associations may be related to preclinical AD. Additionally, we focused on midlife individuals, the ideal cohort for developing primary or secondary prevention strategies. Thirdly, we utilized high-resolution brain scans for all participants along with state-of-the-art methods for sampling and quantifying the images, alongside multivariable adjustments for potential confounders such as APOE4 genotype and midlife health variables. Lastly, given the established evidence of sex differences in stress response^30^, possibly mediated by changes in sex steroid hormones, we specifically examined the impact of sex and menopause status on cortisol-biomarker associations.

Present findings indicate possible deleterious effects of cortisol on AD risk already in midlife, as reflected in its associations with Aβ load and select neurodegenerative biomarkers. These effects varied by sex and, in the context of CMRglc, were further differentiated by menopause status. On volumetric MRI, cortisol exhibited negative associations with total brain volume for both genders, with some regional variations, where men exhibited stronger associations in parietal regions, while women showed more pronounced effects in frontal areas. These data add to previous evidence of stronger associations of cortisol with reduced brain volume in frontal regions of midlife women compared to age-controlled men^23^. The identified male-specific effects in parietal regions warrant further investigation. Alike the previous MRI study of midlife individuals^23^, we found no clear associations between cortisol levels and hippocampal volume. This suggests that the associations of cortisol with brain volume in midlife may not be specific to hippocampus but diffuse throughout the gray matter. Given that hippocampal atrophy has been linked with hypercortisolism, and that some studies of elderly, MCI and AD patients reported associations between cortisol and hippocampal volume^20,22^, it remains to be established whether cortisol effects on hippocampal morphology become more pronounced with advancing age or in presence of neurodegenerative disease. It is also possible that changes are present, such as subregional hippocampal atrophy or synaptic loss, but not detectable with our analytic methods.

Nonetheless, we observed sex-specific associations between cortisol and PCr/ATP in the cluster combining posterior cingulate, frontal and temporal regions, including medial temporal lobe. This pattern was driven by the male group exhibiting stronger associations than the female group. On the other hand, cortisol generally had neutral or positive associations with CMRglc in men, while it demonstrated negative associations in women, especially in frontal regions. These data suggest sex differences in brain metabolic responses to stress. In men, the positive association with PCr/ATP suggests that higher cortisol might lead to either a reduction in ATP production or an increase in ATP consumption. This could be indicative of a stress response where energy reserves (PCr) are maintained or increased to rapidly replenish ATP during acute demands. This hypothesis is supported by the neutral or positive cortisol-CMRglc associations observed in men, potentially reflecting preserved or increased glucose uptake. Further, both cortisol levels and cortisol-associated PCr/ATP ratios in frontotemporal regions were linked to lower memory performance in men, suggesting direct and indirect adverse effects of stress on memory. Conversely, the negative association of cortisol with CMRglc, along with the lack of association with PCr/ATP observed in women, suggest that women’s brains might either have more efficient ATP production or utilization under stress, or respond to cortisol in a manner that doesn't significantly alter the PCr/ATP balance. It is possible that elevated cortisol levels in women led to reduced glucose demand or utilization, possibly as a protective mechanism against stress-induced metabolic demands, or that women’s brains might sustain ATP production through other pathways, such as increased ATP synthesis or use of alternative energy substrates such as ketone bodies^26^. This aligns with evidence of sex-specific molecular and energetic responses to stress across the lifespan^30^, alongside preclinical findings of higher ATP levels and increased mitochondrial function in female mice compared to males^35^ and clinical evidence of lower ^31^P-MRS PCr/ATP ratios, reflecting higher ATP utilization, in midlife women compared to age-controlled men^36,37^. Notably, in women, we observed positive associations between PCr/ATP levels in precuneus and parietal regions and memory scores, alongside a lack of significant cortisol impact on memory. This may reflect a compensatory reaction. It is well-documented that women generally outperform men in various cognitive domains, a trend that persists after a dementia diagnosis^38^ and despite exhibiting more severe AD pathology^39^. Herein, associations of cortisol with Aβ load were more pronounced among women, suggesting an earlier pathophysiological effect.

Overall, the present study suggests that men may be more susceptible to the effects of cortisol on cortical volume and ATP production, whereas women may be more vulnerable to cortisol-associated glucose dysregulation and Aβ deposition. These differences may stem from biological and hormonal variations. For instance, estrogen decline during menopause affects brain bioenergetics^26^, and may disrupt cellular processes involved in HPA axis activation and feedback^30^. In the present study, men exhibited stronger associations between cortisol levels and PCr/ATP ratios compared to both postmenopausal and perimenopausal groups, whereas the postmenopausal group, but not the perimenopausal group, showed negative associations between cortisol and CMRglc in frontal regions in contrast to men, who exhibited non-significant, generally positive associations. We and others have shown that postmenopausal and to a lesser extent, perimenopausal women exhibit lower CMRglc^40–46^ and PCr/ATP levels^36,37,42^, as well as greater neuropathological burden^40–43,47,48^ compared to premenopausal women and age-controlled men. Current findings suggest a female-specific metabolic response to cortisol that become more pronounced postmenopause, which warrant further investigation.

Limitations of this study include its cross-sectional nature, which precludes investigation of causality or to track dynamic changes in cortisol-biomarker trajectories. Consequently, there is a need for cautious interpretation of the interaction effects observed, underscoring the importance of further validation through replication. Studies with longitudinal follow-ups are needed to explore whether cortisol-related biomarker changes are predictive of AD differently across genders. Further research is also needed to further evaluate cortisol as a preclinical marker of AD risk, and to test the efficacy of HPA-based interventions, such as GR modulators and lifestyle stress-reduction techniques, for AD prevention. Although our analysis controlled for several possible confounders—including age, APOE4 status, midlife health risks, and hormone therapy use—more work is needed to assess the impact of lifestyle and environmental factors on the complex interplay between cortisol and biomarkers of AD risk.

We did not observe reduced cognitive performance in women compared to men, and only men exhibited negative, albeit marginal, associations between cortisol and memory performance. As such, our study did not confirm previous findings of stronger associations of cortisol with memory in women compared to age-controlled men^23^. However, the fact that our participants were generally in good physical health and highly educated may have limited our ability to detect detrimental effects of cortisol on cognition. This suggests that a broader range of cognitive tests or larger sample sizes might be necessary to capture more subtle cognitive changes in relation to cortisol levels. Despite this limitation, we observed significant associations between cortisol and AD biomarkers, both overall and by gender. This suggests that biomarker measures may be more sensitive indicators than cognitive tests for detecting the impact of chronic stress on preclinical AD risk.

Some differences among published studies may be related to differences in cortisol measurement methods (blood vs. saliva or urine) or the use of morning instead of evening cortisol samples. One study found associations between evening serum cortisol levels and both poorer cognitive performance and reduced hippocampal volume in patients with arterial disease^19^. In another study of non-demented elderly, evening salivary cortisol correlated with memory scores, whereas morning cortisol was more closely associated with processing speed and executive functioning^18^. We measured morning cortisol levels once for each participant. Therefore, our study may be underestimating cortisol’s effects on imaging and cognitive outcomes, thus conservatively reducing power in detecting significant associations. Additionally, our study design did not include collection of salivary or 24-h urine cortisol, or other dynamic measures of cortisol fluctuations throughout the day, which might better capture long-term cortisol exposure.

We caution that present results were derived from a cohort of generally healthy, well-educated, predominantly white midlife individuals, mostly from middle to high socioeconomic backgrounds. This demographic profile limits the generalizability of our findings to broader populations.

## Conclusion

Elevated serum cortisol levels are associated with AD biomarkers in asymptomatic midlife individuals at risk for AD, with the associations varying by sex, and to a certain degree, menopausal status. These results suggest sex-specific neurophysiological responses to stress in midlife, and support a role for stress reduction strategies in AD prevention.

## Methods

### Participants and data

This is a natural history, non-interventional study of cognitively normal individuals ages 35–65 years, carrying risk factors for late-onset AD such as a family history and/or APOE4 genotype. Participants were recruited at the Weill Cornell Medicine (WCM) Alzheimer’s Prevention Program between 2018 and 2023 by self-referral, flyers, and word of mouth.

Our inclusion and exclusion criteria were previously described^40–43^. Briefly, all participants had Montreal Cognitive Assessment (MoCA) score ≥ 26 and normal cognitive test performance by age and education^40–43^. Exclusion criteria included medical conditions that may affect brain structure or function (e.g., stroke, any neurodegenerative diseases, major psychiatric disorders, hydrocephalus, demyelinating disorders such as Multiple Sclerosis, intracranial mass, and infarcts on MRI), use of psychoactive medications, and contraindications to MR or PET imaging. None of the participants reported using glucocorticoid medications. All participants received medical, neurological, laboratory, cognitive exams, volumetric MRI and ^31^P-MRS scans within 6 months of each other. Approximately half of our participants also receive PET imaging. A family history of late-onset AD was elicited using standardized questionnaires^40–43^. APOE4 genotype was determined using standard qPCR procedures. Participants carrying one or two copies of the APOE4 allele were grouped together as APOE4 carriers (APOE4+) and compared to non-carriers (APOE4−).

The patients’ sex was determined by self-report. Our study protocol involves a 1:3 enrollment ratio of men to women, with approximately equal representation of premenopausal, perimenopausal, and postmenopausal statuses among women (1:1:1 ratio). Determination of menopausal status was based on the Stages of Reproductive Aging Workshop (STRAW) criteria^49^ with hormone assessments as supportive criteria^44^. Participants were classified as premenopausal (regular cycler), perimenopausal (irregular cyclers with interval of amenorrhea ≥ 60 days or ≥ 2 skipped cycles) and postmenopausal (no cycle for ≥ 12 months)^44^. Information on hysterectomy/oophorectomy status and hormone therapy (HT) usage was obtained through review of medical history.

### Standard protocol approvals, registrations, and patient consents

All methods were carried out in accordance with relevant guidelines and regulations. All assessments and imaging procedures were approved by the Weill Cornell Medicine Institutional Review Board and Radioactive Drug Research Committee. Written informed consent was obtained from all participants.

### Cortisol assessment

Participants received a blood draw by venipuncture between 8 and 9am after an overnight fast. Blood samples were centrifuged and shipped overnight to CLIA-certified Boston Heart Diagnostics (Framingham, MA). Serum cortisol concentration (µg/dL, micrograms per deciliter) was measured with Electrochemiluminescence Immunoassay (ECLIA) on a Roche Cobas e601/e602 analytical unit for immunoassay tests using Electrochemiluminescence technology (ECL) [Roche Diagnostics; Basel, Switzerland], with intra-assay coefficients of variation ranging from 1.8 to 7.1% for high concentrations and low concentrations. The limits of detection of the test were 0.054–63.4 μg/dL.

### Cognitive measures

Participants underwent a cognitive testing battery^40–43^, including Rey Auditory Verbal Learning Test (RAVLT) immediate and delayed recall, Wechsler Memory Scale Logical Memory immediate and delayed recall, Trail Making Test (TMT) B, FAS, animal naming, and Boston object naming. Higher scores across all cognitive endpoints indicate better performance, except for TMT, for which higher scores indicate slower task completion. Cognitive scores were standardized prior to analysis.

### Brain imaging

#### Image acquisition

All participants received a 3D volumetric T_1_-weighted MRI scan on a 3.0 T GE MR 750 Discovery scanner (General Electric, Waukesha, WI) [BRAVO; 1 × 1 × 1 mm resolution, 8.2 ms repetition time (TR), 3.2 ms echo time (TE), 12° flip angle, 25.6 cm field of view (FOV), 256 × 256 matrix with ARC acceleration] using a 32-channel head coil, using published protocols^40–43^. PET scans were acquired on a Siemens BioGraph mCT 64-slice PET/CT operating in 3D mode [70 cm transverse FOV, 16.2 cm axial FOV] following standardized procedures^40–43^. Summed images were obtained 40–60 min post-injection of 5 mCi of ^18^F-fluoro-deoxyglucose (FDG), and 60–90 min post-injection of 15 mCi of ^11^C-Pittsburgh Compound B (PiB). All images were corrected for attenuation, scatter and decay.

The ^31^P-MRS scan was acquired on the same scanner as the MRI, typically on the same day, using a dual tuned 32-channel ^31^P/^1^H quadrature head coil (Clinical MR Solutions, Brookfield, WI) [2048 points, 5000 Hz sweep width, 2000 ms TR, 2 averages, 55° flip angle at 51.3 MHz, 24 cm FOV]^36,37,42^. A 3 Plane Localizer image with 20 images in each orthogonal direction was acquired. Shimming was performed using a ^1^H single voxel technique placed over the entire brain. A high-resolution, 8-slice sagittal T_2_-Fluid Attenuated Inversion Recovery sequence [FLAIR; 2200 ms TR, 12 ms TE, 780 ms inversion time (TI), 24 cm FOV, 0.94 × 0.94 mm] was acquired with a 5 mm slice thickness at the same location as each of the ^31^P-MRS CSI slices for reference. MRS data were processed using XSOS written in IDL^36,37,42^. Raw data was processed using Hamming and Fermi k-space filters, 20 Hertz exponential filtering and zero-filling in time, x and y-domains prior to 3D Fast Fourier Transformation. This resulted in a 16 × 16 image of 1.5 × 1.5 × 3.0 cm voxels with the signal intensity in each voxel corresponding to the peak area of the ^31^P metabolite. Peak area integration was performed around each of four well-resolved resonance peaks: phosphocreatine (PCr), α-ATP, β-ATP and γ-ATP moieties. The PCr peak was set at 0.0 ppm and susceptibility corrections performed by an experienced analyst (JPD). The ratio of PCr over total ATP (sum of α-, β- and γ-ATP) was computed.

#### Multiparametric image analysis

The 3D T_1_-weighted BRAVO MRI scans were first processed in Statistical Parametric Mapping (SPM8)^50^ implemented in Matlab 2021 (MathWorks; Natick, MA). For each participant, we used the Normalized Mutual Information routine of SPM8^50^ to align the T_1_ BRAVO sequence to each PET scan, and to the reference T_2_-FLAIR acquired at exactly the same location as the ^31^P-MRS CSI slices. The parametric metabolite MRS maps were then aligned with the skull stripped 3D T_1_-weighted FreeSurfer scan. Volumetric MRI scans were resampled to a 256 × 256 × 256 matrix array whereas the parametric metabolite MRS maps were resized to 256 × 256 images but not interpolated beyond the original 16 × 16 × 8 matrix given partial volume errors would occur. The co-registered images were quantified using the subcortical gray and white matter segmentation tools implemented in FreeSurfer 6.0^51^ running under the Centos 7 Linux environment and Desikan-Killiany Atlas-based regions of interest (ROI)^52^ applied to the aligned MRI for regional sampling.

We focused on ROIs with known AD vulnerability^31^: medial temporal lobe (hippocampus, amygdala, entorhinal and parahippocampal gyrus), middle and superior frontal gyrus; posterior cingulate gyrus and precuneus; inferior, middle and superior temporal gyrus; and inferior and superior parietal gyrus. Hippocampus was examined in analysis of MRI volume, whereas the medial temporal ROI was used for MRS and FDG-PET due to their lower resolution. For PiB analysis, we created an AD-mask by averaging parietal, temporal, frontal, posterior cingulate and precuneus ROIs. Biomarker data were normalized by modality-specific confounders: MRI volumes were adjusted by total intracranial volume; ^31^P-MRS ATP measures were normalized to PCr; FDG- and PiB-PET measures were normalized to cerebellar gray matter uptake obtained with FreeSurfer to derive standardized uptake value ratios (SUVR).

### Clinical covariates

In Model 1, all analyses were adjusted by age, sex, and APOE-4 status. Cognitive analyses were also adjusted by years of education. In Model 2, we further examined midlife health variables, including smoking (self-reported as ever vs. never smoker), hypertension (systolic BP ≥ 140 mmHg, a diastolic BP ≥ 90 mm Hg, or self‐reported antihypertensive medication use), hypercholesterolimia (total cholesterol ≥ 240 mg/dL or self-reported use of cholesterol-lowering medications), diabetes (fasting glucose level ≥ 126 mg/dL or self‐reported use of glucose-lowering medications), and depression (Hamilton test and/or the Patient-Reported Outcomes Measurement Information System (PROMIS) Depression measure or self-reported use of antidepressant medications). For women, we also examined menopausal status (postmenopausal vs. pre/perimenopausal) and HT use (user vs. never-user).

### Statistical analysis

Analyses were performed in R v.4.2.0 and SPSS version 28. Clinical measures were examined using general linear models or chi-squared tests, as appropriate. As cortisol and PiB SUVR measures did not follow a Gaussian distribution, we used the bestNormalize R package to identify the optimal normalization transformation for each measure. The standardized asinh(x) transformation was selected for cortisol and AD-mask SUVR, and the OrderNorm transformation for regional PiB SUVR values. Following the transformations, all variables passed the Shapiro Wilks test for normality and their histograms displayed normal distributions.

Our primary outcome for PiB-PET was Aβ load in AD-mask. For the other modalities, we used Principal component analysis (PCA)^53^ to reduce the dimensionality from the original 10 ROIs across each imaging modality, using varimax rotation with kaiser normalization. An eigenvalue threshold of 1.0 was predetermined to select factors for inclusion in hypothesis testing. PCA extracted distinct factors for each modality: two factors from MRI gray matter volumes, two factors from PCr/ATP, three factors from CMRglc. The composition of the extracted factors is detailed in Supplementary Table 1. These factors accounted for > 68% of the total variance in the biomarker panel for each modality. For all participants, we computed standardized scores for each identified factor, which represent a linear combination of the regional biomarker measures showing substantial loading (coefficients > 0.55) within each respective factor. Our primary analysis focuses on PCA-derived factors, as this approach effectively captures the interrelatedness of regional biomarker data and streamlines the hypothesis testing process. For comparison with previous studies^23^, we also included total brain volume.

Multivariable linear regression models were trained to test for associations between cortisol and each biomarker and cognitive outcome, adjusting by covariates. We then examined sex modification effects by developing linear regression models including multiplicative interaction terms, for all outcomes. Each model contained cortisol, sex, and their interaction as covariates, as well as clinical covariates. Estimates are presented for the overall study sample as well as by sex. The stratified analysis was performed to investigate hypothesized differences in the strength of the correlations by sex and to mitigate the effects of its confounding on the overall correlations. Analyses were performed separately for each biomarker outcome. Results were considered significant at *P* < 0.05. Given that each biomarker was examined independently and the PCA method effectively reduced the dimensionality of our data, correction for multiple comparisons was not performed.

### Sensitivity analysis

#### Associations of cortisol and regional biomarker measures

To examine which regions were involved in the above effects, multivariable linear regressions were used to test for associations between cortisol and sex, and their interactions, in the full ROI panel. Analyses were performed separately for each biomarker outcome. Results are reported at *P* < 0.05. To address the issue of multiple comparisons, we applied the Benjamini–Hochberg correction method, which controls the false discovery rate (FDR) by adjusting the significance threshold to account for intercorrelated measures^54^.

#### Effects of menopausal status

We used multiple linear regressions to test for effects of menopausal status on the associations of cortisol and biomarker outcomes. To reduce the number of comparisons and therefore the potential for Type I error, this analysis was limited to biomarker measures demonstrating significant main effects of sex in their relationship with cortisol levels. As shown in Supplementary Table 4, the postmenopausal group was older than the premenopausal group, while no age difference was found between postmenopausal and perimenopausal groups, or compared to men. Given these considerations and the narrower biomarker range in the premenopausal group, analysis was restricted to age-comparable groups (postmenopausal, perimenopausal, and men). In combination with age correction procedures, this approach ensures a more accurate examination of menopausal status effects. We structured regression models to explore pairwise combinations between groups (postmenopause vs. men; perimenopause vs. men), with each model containing cortisol, sex, and their interaction, and clinical covariates. Results were considered significant at *P* < 0.05.

#### Mediation analysis

We used multiple linear regressions to test for mediation effects by examining associations between cortisol-related regional biomarkers and cognitive measures, at *P* < 0.05. To reduce the number of comparisons and therefore the potential for Type I error, this analysis was limited to cognitive measures demonstrating significant sex modification effects in their relationship with cortisol levels.

### Supplementary Information

Supplementary Tables.

## Data Availability

De-identified source data files and statistical code will be made available to qualified investigators for the purpose of replicating procedures and results upon reasonable request to the corresponding author.
