# Supplementary material for: Sex-specific associations of serum cortisol with brain biomarkers of Alzheimer’s risk
Source: Sci Rep. 2024 Mar 6;14:5519. doi: 10.1038/s41598-024-56071-9 (PMC10918173; doi:10.1038/s41598-024-56071-9)
Supplement: Supplementary file 1 — Supplementary Tables. [file 41598_2024_56071_MOESM1_ESM.docx]

## Supplementary Table 1. Principal component analysis: factor structure and variance explained

|  | Gray matter volume | | Phosphocreatine (PCr)/ adenosine triphosphate (ATP) | | Glucose metabolic rates | | |
| --- | --- | --- | --- | --- | --- | --- | --- |
|  | Component 1 | Component 2 | Component 1 | Component 2 | Component 1 | Component 2 | Component 3 |
| Inferior parietal | 0.464 | **0.630** | 0.322 | **0.879** | **0.938** | 0.070 | 0.265 |
| Inferior temporal | **0.630** | 0.464 | **0.809** | 0.354 | **0.726** | 0.629 | -0.011 |
| Medial temporal* | **0.717** | 0.175 | **0.877** | 0.217 | -0.101 | **0.934** | 0.110 |
| Middle frontal | **0.766** | 0.398 | **0.829** | 0.166 | 0.405 | 0.569 | **0.661** |
| Middle temporal | **0.730** | 0.328 | **0.833** | 0.410 | 0.324 | **0.864** | 0.283 |
| Posterior cingulate | **0.657** | 0.290 | **0.670** | 0.448 | **0.638** | 0.374 | 0.539 |
| Precuneus | 0.384 | **0.841** | 0.208 | **0.946** | **0.939** | 0.055 | 0.275 |
| Superior frontal | **0.777** | 0.340 | **0.801** | 0.209 | 0.317 | **0.759** | 0.510 |
| Superior temporal | **0.766** | 0.274 | **0.898** | 0.294 | 0.208 | **0.922** | 0.156 |
| Superior parietal | 0.215 | **0.898** | 0.328 | **0.914** | **0.935** | 0.203 | 0.083 |

Factor interpretation is based on the strongest loading coefficients within each matrix component, or factor. For example, for gray matter volume, Factor 1 includes mainly frontal and temporal regions, and Factor 2 includes mainly parietal regions and precuneus (coefficients >0.63 in each factor). Dominant regions within each factor are in bold.

*Hippocampus is included instead of medial temporal lobe for gray matter volume measures.

## Supplementary Table 2. Associations of cortisol with cognitive performance by sex

|  | **Men** | | | **Women** | | |  |
| --- | --- | --- | --- | --- | --- | --- | --- |
| **Model 1** | Coeff. | 95% CI | *P* | Coeff. | 95% CI | *P* | *Interaction P* |
| Logical memory, immediate | -0.178 | -0.720, 0.154 | 0.200 | -0.011 | -0.139, 0.118 | 0.874 | 0.328 |
| Logical memory, delayed | -0.164 | -0.627, 0.163 | 0.244 | 0.045 | -0.088, 0.174 | 0.518 | 0.259 |
| RAVLT, total | -0.286 | -0.846, -0.017 | **0.042** | -0.009 | -0.137, 0.121 | 0.903 | **0.046** |
| RAVLT, delayed | -0.358 | -0.909, -0.127 | **0.010** | -0.066 | -0.191, 0.066 | 0.338 | 0.085 |
| RAVLT, recognition | -0.097 | -0.642, 0.311 | 0.489 | -0.080 | -0.193, 0.052 | 0.299 | 0.964 |
| FAS | 0.239 | -0.067, 0.727 | 0.102 | -0.031 | -0.166, 0.106 | 0.660 | 0.091 |
| TMT-B | -0.080 | -1.065, 0.618 | 0.595 | -0.090 | -0.105, 0.024 | 0.212 | 0.229 |
| **Model 2** |  |  |  |  |  |  |  |
| Logical memory, immediate | -0.207 | -0.771, 0.114 | 0.142 | -0.0218 | -0.146, 0.113 | 0.802 | 0.283 |
| Logical memory, delayed | -0.174 | -0.654, 0.163 | 0.234 | 0.0235 | -0.098, 0.164 | 0.616 | 0.223 |
| RAVLT, total | -0.318 | -0.895, -0.064 | **0.025** | -0.018 | -0.146, 0.112 | 0.796 | **0.035** |
| RAVLT, delayed | -0.376 | -0.953, -0.136 | **0.010** | -0.076 | -0.188, 0.070 | 0.370 | 0.074 |
| RAVLT, recognition | -0.101 | -0.677, 0.333 | 0.496 | -0.053 | -0.173, 0.078 | 0.453 | 0.887 |
| FAS | 0.219 | -0.100, 0.696 | 0.138 | -0.024 | -0.160, 0.112 | 0.732 | 0.128 |
| TMT-B | -0.093 | -1.073, 0.548 | 0.517 | -0.094 | -0.108, 0.023 | 0.199 | 0.228 |

Standardized beta coefficients and 95% confidence intervals (CI) from regression models adjusted by age, education, and APOE4 status (Model 1); and after further adjustment by midlife health variables for both genders, and by hormone therapy use for women (Model 2). Cognitive test scores are standardized. Significant P values are in bold.

Abbreviations: RAVLT, Rey Visual Auditory Verbal Learning test; TMT-B, trail making test-B

## Supplementary Table 3. Associations of cortisol with regional gray matter volume by sex

|  | **Men** | | | **Women** | | |  |
| --- | --- | --- | --- | --- | --- | --- | --- |
| **Gray matter volume** | Coeff. | SE | *P* | Coeff. | SE | *P* | *Interaction P* |
| Hippocampus | 11.70 | 76.97 | 0.880 | -12.45 | 19.36 | 0.521 | 0.613 |
| Inferior parietal | -578.65 | 285.41 | **0.048** | -41.96 | 85.17 | 0.623 | 0.086 |
| Inferior temporal | -297.80 | 257.91 | 0.254 | -31.18 | 68.31 | 0.649 | 0.191 |
| Middle frontal | -89.41 | 208.38 | 0.670 | -135.61 | 55.52 | **0.015** | 0.825 |
| Middle temporal | 158.82 | 236.93 | 0.506 | -125.38 | 71.81 | 0.082 | 0.144 |
| Posterior cingulate | -120.06 | 95.37 | 0.214 | -11.89 | 23.25 | 0.610 | 0.225 |
| Precuneus | -359.07 | 185.86 | 0.060 | -51.67 | 49.32 | 0.296 | 0.229 |
| Superior frontal | -237.28 | 419.48 | 0.574 | -0.440 | 99.01 | 0.996 | 0.360 |
| Superior temporal | -198.79 | 244.53 | 0.420 | -45.57 | 62.29 | 0.446 | 0.608 |
| Superior parietal | -631.50 | 289.45 | **0.034** | -25.61 | 78.97 | 0.746 | 0.051 |
| **PCr/ATP** |  |  |  |  |  |  |  |
| Inferior parietal | 0.126 | 0.076 | 0.106 | 0.006 | 0.018 | 0.728 | **0.026** |
| Inferior temporal | 0.085 | 0.035 | **0.018*** | 0.006 | 0.009 | 0.527 | **0.012*** |
| Medial temporal | 0.348 | 0.146 | **0.021** | 0.017 | 0.036 | 0.640 | **0.011*** |
| Middle frontal | 0.134 | 0.060 | **0.030** | -0.016 | 0.016 | 0.313 | **0.006*** |
| Middle temporal | 0.080 | 0.038 | **0.042** | 0.006 | 0.009 | 0.499 | **0.022** |
| Posterior cingulate | 0.229 | 0.064 | **<0.001*** | 0.005 | 0.015 | 0.748 | **<0.001*** |
| Precuneus | 0.102 | 0.062 | 0.107 | 0.035 | 0.019 | 0.070 | 0.144 |
| Superior frontal | 0.202 | 0.062 | **0.002*** | -0.012 | 0.018 | 0.518 | **<0.001*** |
| Superior temporal | 0.087 | 0.038 | **0.027** | 0.000 | 0.010 | 0.970 | **0.008*** |
| Superior parietal | 0.145 | 0.085 | 0.096 | 0.0248 | 0.021 | 0.185 | 0.054 |
| **CMRglc** |  |  |  |  |  |  |  |
| Inferior parietal | 0.045 | 0.024 | 0.082 | -0.012 | 0.017 | 0.471 | 0.316 |
| Inferior temporal | 0.033 | 0.018 | 0.090 | -0.003 | 0.010 | 0.730 | 0.583 |
| Medial temporal | 0.014 | 0.012 | 0.269 | -0.009 | 0.008 | 0.274 | 0.491 |
| Middle frontal | 0.085 | 0.029 | **0.012*** | -0.037 | 0.014 | **0.050** | **0.023** |
| Middle temporal | 0.028 | 0.018 | 0.132 | -0.023 | 0.011 | 0.064 | 0.141 |
| Posterior cingulate | 0.031 | 0.021 | 0.166 | -0.016 | 0.012 | 0.188 | 0.277 |
| Precuneus | 0.065 | 0.030 | **0.046** | -0.012 | 0.018 | 0.507 | 0.318 |
| Superior frontal | 0.074 | 0.030 | **0.029** | -0.029 | 0.014 | **0.036** | **0.028** |
| Superior temporal | 0.033 | 0.011 | **0.012** | -0.0135 | 0.011 | 0.155 | 0.219 |
| Superior parietal | 0.050 | 0.031 | 0.136 | -0.015 | 0.014 | 0.303 | 0.266 |
| **Amyloid-β load** |  |  |  |  |  |  |  |
| Inferior parietal | -0.084 | 0.109 | 0.447 | 0.030 | 0.048 | 0.531 | 0.428 |
| Inferior temporal | 0.127 | 0.108 | 0.247 | 0.018 | 0.047 | 0.696 | 0.600 |
| Middle frontal | 0.112 | 0.100 | 0.269 | 0.036 | 0.047 | 0.444 | 0.596 |
| Middle temporal | -0.002 | 0.104 | 0.981 | 0.071 | 0.047 | 0.131 | 0.595 |
| Posterior cingulate | -0.019 | 0.116 | 0.873 | 0.054 | 0.048 | 0.265 | 0.754 |
| Precuneus | -0.171 | 0.134 | 0.209 | 0.008 | 0.047 | 0.871 | 0.204 |
| Superior frontal | 0.098 | 0.107 | 0.429 | 0.017 | 0.047 | 0.724 | 0.382 |
| Superior temporal | 0.041 | 0.116 | 0.729 | 0.051 | 0.046 | 0.268 | 0.802 |
| Superior parietal | -0.101 | 0.174 | 0.564 | -0.036 | 0.046 | 0.437 | 0.732 |

Unstandardized beta coefficients and standard errors (SE) from regression models adjusted by age, education, APOE4 status, and midlife health variables for both sexes, and by hormone therapy use for women. All analyses are adjusted by modality-specific confounders. ^31^P-MRS measures are phosphocreatine (PCr) to adenosine triphosphate (ATP) ratios. ^18^F-FDG PET cerebral metabolic rates of glucose (CMRglc) are standardized uptake value ratios (SUVR) to cerebellar gray matter uptake. ^11^C-PiB PET measures are standardized uptake value ratios (SUVR) to cerebellar gray matter uptake.

## Significant *P* values are in bold; *Significant after Benjamini-Hochberg correction for multiple comparisons.

## Supplementary Table 4. Participant characteristics by menopause status

|  | Overall | Men | Women | | |
| --- | --- | --- | --- | --- | --- |
|  |  |  | Premenopause | Perimenopause | Postmenopause |
| N | 277 | 57 | 57 | 75 | 88 |
| Cortisol, μg/dL | 11.1(4.7) | 11.0(3.0) | 9.7(3.6) | 11.2(5.7) | 11.9(4.6) |
| Age, years, range | 51(7) | 51(3) | 45(4), 40-52 | 49(4), 42-65 | 56(5)^, 41-65 |
| Education, years | 17(2) | 18(2) | 17(2) | 17(2) | 17(2) |
| MoCA scores, unitless | 28(2) | 29(1) | 28(2) | 28(2) | 29(2) |
| Race, % white | 81 | 75 | 75 | 85 | 84 |
| APOE-4 status, % carrier | 44 | 53 | 39 | 46 | 43 |
| Smoking, % ever | 22 | 19 | 26 | 24 | 24 |
| Hypertension, % | 9 | 19 | 4* | 7* | 7* |
| Diabetes, % | 2 | 5 | 0 | 1 | 2 |
| Hypercholesterolemia, % | 7 | 7 | 8 | 3 | 8 |
| Depression history, % | 16 | 12 | 18 | 17 | 17 |
| Oophorectomy status, % | - | - | - | - | 19 |
| Menopause hormone therapy, % user | - | - | - | 23 | 34 |

Mean (standard deviation) unless otherwise specified.

**P* < 0.05, different from male group; ^*P* < 0.05, different from premenopausal group

Abbreviations: APOE-4, Apolipoprotein E (APOE) epsilon 4; MoCA, Montreal Cognitive Assessment

## Supplementary Table 5. Associations of cortisol with biomarkers by sex and menopause status

|  | Men | | | POST | | | PERI | | | Men vs. POST | Men vs. PERI |
| --- | --- | --- | --- | --- | --- | --- | --- | --- | --- | --- | --- |
|  | Coeff. | SE | P | Coeff. | SE | P | Coeff. | SE | P | P_interaction_ | P_interaction_ |
| Brain volume FAC2_parietal_ | -0.322 | 0.204 | **0.017** | -0.048 | 0.102 | 0.637 | 0.004 | 0.080 | 0.957 | 0.063 | 0.092 |
| PCr/ATP FAC1_frontotemporal_ | 0.379 | 0.223 | **0.004** | -0.169 | 0.108 | 0.123 | 0.001 | 0.093 | 0.990 | **0.001** | **0.009** |
| CMRglc FAC3_middlefrontal_ | 0.548 | 0.195 | 0.054 | -0.194 | 0.092 | **0.039** | -0.082 | 0.080 | 0.310 | **0.024** | 0.179 |

Standardized beta coefficients and standard errors (SE) from regression models adjusted by age, APOE4 status, midlife health variables, and hormone therapy use. Significant *P* values are in bold.

Brain volumes are adjusted by total intracranial volume. ^31^P-MRS measures are phosphocreatine (PCr) to adenosine triphosphate (ATP) ratios. Cerebral metabolic rates of glucose (CMRglc) measures are standardized to cerebellar gray matter uptake.

Abbreviations: PERI, perimenopausal; POST, postmenopausal.
